# Effects of varying group sizes on performance, body defects, and productivity in broiler chickens

**DOI:** 10.5194/aab-65-171-2022

**Published:** 2022-05-05

**Authors:** Musa Sarıca, Koray Karakoç, Kadir Erensoy

**Affiliations:** Department of Animal Science, Agricultural Faculty, Ondokuz Mayis University, Samsun, Turkey

## Abstract

This study aimed to determine the changes in the performance, welfare, and productivity level of broiler chickens reared at various group sizes (GS3000, GS4000, GS6000, and GS20 000) under intensive field conditions. The study was carried out according to a randomized block design with four different group sizes (GS) in three trials. Weekly body weights (BWs) were determined randomly in 150 individuals from each GS group. Feed intake (FI), feed conversion ratio (FCR), and European production efficiency factor (EPEF) were determined for each GS treatment. Body defects (footpad dermatitis, FPD, hock burn, HB, and the breast burn, BB) were measured randomly in 150 chickens (75 male and 75 female) from each group using a visual scoring system with a 0–3 scale. At 1 and 2 weeks of age, GS3000 broilers had similar BW to GS6000 and higher than GS4000 and GS20 000. However, this situation changed at 6 weeks of age and the male chickens in GS6000 became heavier than in GS3000, GS4000 and GS20 000 ( P = 0.007). No differences in mean values of temperature, humidity, air velocity and litter moisture levels were observed among GS treatments. GS3000 and GS4000 chickens had significantly lower levels of FPD, HB, and BB than chickens reared in GS6000 and GS20 000 ( P < 0.001). The EPEF values from highest to lowest were 425.8, 404.5, 358.8, and 354.0 in the GS6000 GS3000, GS4000, and GS20 000 groups, respectively. In conclusion, our study results showed that rearing in groups of 6000 broilers had both better performance and higher overall productivity than other groups but tended to show more severe body defects.

## 1 Introduction

Broiler chicken production is one of the most crucial animal protein sources for human nutrition, and approximately 7 billion chickens were slaughtered worldwide in 2018 (FAOSTAT, 2020). Improved genetics and breeding techniques account for approximately 80 % to 90 % of production levels observed today (Havenstein et al., 2003a, b); housing and management conditions greatly affect the productivity and output of broiler production (Sarıca and Erensayın, 2018). Increased housing capacity and the intensive production model were accompanied by increased stocking density and group sizes. However, unlike intensive production, poultry, like many other animal species, tend to live in groups or flocks in nature (Collias and Collias, 1996; Christman and Lewis, 2005). The position of individuals in a group in the wild may have benefits related to preventing danger from the outside environment, access to resources (e.g., feed and water), or reduced antagonistic relationships with animals in other groups (Hemelrijk, 2000). Group size and density can affect the performance, welfare, social behavior, movement, and spatial use of chickens (Estevez 2007; Estevez et al., 2007; Averos and Estevez, 2018), which can cause social and physical restrictions in chicken activity (Grigor et al., 1995). Feed efficiency worsened with larger group size, while smaller flock size increases livability (Tind and Ambrosen, 1988). In broiler chickens reared in small and medium group sizes, the slaughter weight and weekly live weight gain have been shown to be higher than in large flock sizes (Ghosh et al., 2012; El-Tahawy et al., 2017). Moreover, increased stocking density and group size increase competition among animals, which causes psychological and physiological stress that negatively affects the welfare of chickens. Hock burn, breast blisters, and especially footpad dermatitis are known welfare problems that can cause painful issues and reduced efficiency for broiler chickens (Bradshaw et al., 2002; Buijs et al., 2009; Hepworth et al., 2010). The development of foot pad dermatitis, hock, and breast burn lesions seems to be similar, and they are a form of contact dermatitis that affects the skin areas that come into contact with unsuitable or irritating substances (Greene et al., 1985; Haslam et al., 2007). Superficial lesions appear as discolored areas and mild hyperkeratosis of the skin of the foot pad and hocks, which can progress to deep ulcers of the skin and necrosis of the epidermis and inflammation of the subcutis (Michel et al., 2012). The foot pads are most commonly affected, followed by the hocks and breast skins (Greene et al., 1985).

Intensive production is widely used for broiler production in large housing capacities ranging from 10 000 to 40 000 birds (Prabakaran, 2003; El-Tahawy et al., 2017). In this type of production system, the hypothesis is that grouping may increase performance, body defects, and productivity in broilers. Most of the studies on group size are generally carried out under trial conditions and with limited chicken populations. However, conducting this study under field conditions is considered important in terms of its effectiveness and applicability. This study aimed to reveal changes in the performance, welfare, and productivity level of broiler chickens reared at various group sizes under intensive field conditions.

**Table 1:** Specifications of houses and trial designs ${}^{*}$ .

Specifications	Trial 1		Trial 2		Trial 3	
	6000	20 000	4000	20 000	3000	20 000
Floor space ( $m^{2}$ )	810	1080	810	1080	810	1080
House capacity (chickens)	12 000	20 000	12 000	20 000	12 000	20 000
Number of groups	2	1	3	1	4	1
Floor space of groups ( $m^{2}$ )	405	1080	270	1080	202.5	1080
Chickens in each group	6000	20 000	4000	20 000	3000	20 000
Stocking density ( $\mathrm{birds}\;m^{-2}$ )	14.8	18.5	14.8	18.5	14.8	18.5
% of thinned chickens	–	33.4	–	10.3	–	21.1
Age at thinning (days)	–	32	–	32	–	34
Age at slaughter (days)	39	39	41	41	39	39

${}^{*}$ Group sizes of 6000, 4000, and 3000 chickens were tested in Trials 1, 2, and 3, respectively, and a group of 20 000 chickens was also tested in each trial.

## 2 Materials and methods

### 2.1 Trial design

Three trials were conducted from October to November 2019 (Trial 1: GS6000 and GS20 000), December 2019 to January 2020 (Trial 2: GS4000 and GS20 000), and February to March 2020 (Trial 3: GS3000 and GS20 000). The study was carried out according to a randomized block design with four different group sizes in three trials. Each trial was performed simultaneously in two separate houses. Two treatments were tested as a concept in all trials. One of the poultry houses was divided into groups of varying sizes in each trial (GS3000, GS4000, and GS6000). Rearing was applied as a single flock in the other house without grouping (GS20 000). Additionally, thinning was applied to GS20 000 chickens at ages of 32, 32, and 34 d in the first, second, and third trials, respectively. Summary information for the trial designs is provided in Table 1.

In the first trial, the GS6000 (group size of 6000 chicks in each of the two replicates) and the GS20 000 (non-grouped single flock) under intensive conditions were compared. The GS6000 house was divided equally into two groups (pen dimensions for each group: 30 m × 13.5 m ). A total of 6000 chicks were placed in each group (405 $m^{2}$ ) for a stocking density of 14.8 $\mathrm{birds}\;m^{-2}$ . When the first trial was terminated, slaughtering was performed at 39 d .

In the second trial, the GS4000 (group size of 4000 chicks in each of the three replicates) and the GS20 000 (non-grouped single flock) under intensive conditions were compared. The GS4000 house was divided into three equal areas of 270 $m^{2}$ , and 4000 chickens were reared at a stocking density of 14.8 $\mathrm{birds}\;m^{-2}$ per group. The second trial ended with the transfer of chickens at 41 d to a commercial slaughterhouse.

In the third trial, the GS3000 (group size of 3000 chicks in each of the four replicates) and the GS20 000 (non-grouped single flock) under intensive conditions were compared. The GS3000 house was divided into four equal groups, each with a floor area of 202.5 $m^{2}$ . In this trial, 3000 chickens were reared at a 14.8 $\mathrm{birds}\;m^{-2}$ stocking density in each group. The third trial ended with the transfer of chickens to a slaughterhouse at 39 d .

In the GS20 000 house, classic intensive rearing was applied without grouping the whole house during each of the three trials. A total of 20 000 chicks were placed in a 1080 $m^{2}$ area, resulting in a stocking density of 18.5 $\mathrm{birds}\;m^{-2}$ . Additionally, in Trials 1, 2, and 3, GS20 000 chickens were thinned by 33.4 %, 10.3 %, and 21.1 %, at 32, 32, and 34 d , respectively. GS20 000 chickens were slaughtered at 39, 41, and 39 d , the same as the other groups (GS3000, GS4000, and GS6000) in the first, second, and third trial, respectively.

**Table 2:** Nutritional values of feeds used in the trials.

Nutrients	Broiler chicken starter	Broiler chicken	Broiler chicken
	(1 to 11 d )	(11 to 21 d )	(21 to SA ${}^{*}$ )
Crude protein (%)	23	22	20
Metabolizable energy ( $\mathrm{kcal}\;{\mathrm{kg}}^{-1}$ )	3000	3050	3150
Crude cellulose (%)	4.0	4.0	4.0
Crude ash (%)	7.0	6.0	6.0
Calcium (%)	1.10	1.00	0.90
Phosphorus (%)	0.65	0.60	0.55
Methionine (%)	0.50	0.45	0.45
Lysine (%)	1.30	1.20	1.20

${}^{*}$ SA – slaughter age (chickens were slaughtered at 39, 41, and 39 d in Trials 1, 2, and 3, respectively).

Ross-308 broiler chicks, which were obtained from a commercial hatchery at 1 d , were distributed to both houses according to the trial design. Both houses were built similarly but in different capacities (12 000 bird capacity house for GS3000, GS4000, and GS6000; 20 000 bird capacity house for GS20 000) and were environmentally controlled. The climate was regulated automatically using ventilation controlled mechanically by fans, while the lighting was provided artificially. The poultry houses were cleaned, washed, and disinfected after each trial. The floors were concrete, and the litter was rice husk (approximately 8–10 cm thickness) for each trial. All animals were provided with feed and water ad libitum. Feeds were provided using a commercial feed mill, and their nutritional values are provided in Table 2.

Chicks received continuous light during the first 2 d and were then maintained on the following light cycles: 23 h of light and 1 h of dark (age of 3–7 d ); 22 h of light and 2 h of dark (age of 8–21 d ); and 21 h of light and 3 h of dark (age of 22 d to slaughter age). The light intensity was 15–20 lx at a chicken level during each trial. Feeding and drinking were performed using a spiral feeder and nipple drinker system, which are widely used in broiler production, and sufficient equipment was provided for each chicken. Both houses were similar in terms of their structural traits, and only their capacities differed. In the trials, the size of the grouped (multiple flocks) house was 13.5 m × 60 m and the ungrouped (single flock) house was 13.5 m × 80 m . During the production period, all health protection and biosecurity measures were taken. Chicks were obtained from the hatchery with infectious bronchitis (IB) and Newcastle disease (ND) vaccines. Additionally, infectious bursal disease (IBD) and ND vaccinations were performed in the house through the production periods.

### 2.2 Data collection

The same procedures were applied for data collection in all trials. In the GS6000 house, two groups were created. When the chicks were transferred from the hatchery to the house, 150 random chicks were individually weighed, and an equal number of chicks (6000 chicks for each pen) were placed into two pens. These operations were performed for GS4000 chickens (dividing by three equal pens) in the second trial and GS3000 birds (dividing by four equal pens) in the third trial. Weekly body weights (BWs) were determined individually for 150 chickens at random from each group with a 1 g precision scale. Unlike for GS6000, GS4000, and GS6000 chickens, GS20 000 chickens were thinned at different ages, and their slaughter BWs were taken from the slaughterhouse in all trials. Feed intake (FI) and feed conversion ratio (FCR) were determined for the entire production period on a house basis. FCR was measured as in Eq. ([Disp-formula Ch1.E1]).

1 $$\text{FCR}=\frac{\text{feed intake }(g)}{\text{body weight }(g)}$$

The number of dead chickens was determined daily for each trial, and livability (%) is given as in Eq. (2).

2 $$\text{livability}=100-\left(\frac{\text{number of dead chickens}\times 100}{\text{total number of chickens}}\right)$$

European production efficiency factor (EPEF) was used to determine productivity according to Huff et al. (2013) and given as in Eq. (3).

3 $$\text{EPEF}=\left(\frac{\text{livability }(\%)\times \text{ body weight }(\mathrm{kg})\times 100}{\text{feed conversion ratio}\times \text{ trial duration }(d)}\right)$$

**Table 3:** Least square mean and standard error (SE) of weekly body weight changes (grams per week) in broilers reared in varying group sizes ${}^{1}$ .

Ages	GS3000	GS4000	GS6000	GS20 000	${\text{df}}_{\text{gs}}$ ${}^{2}$	F value	P value
Day 0	43.9 ± 0.40	43.6 ± 0.39	43.6 ± 0.38	43.4 ± 0.26	3	0.65	0.582
Week 1	183.3 ± 2.75 ${}^{\text{ab}}$	176.6 ± 2.71 ${}^{b}$	184.4 ± 2.60 ${}^{a}$	178.5 ± 1.67 ${}^{b}$	3	2.81	0.040
Week 2	544.6 ± 10.54 ${}^{a}$	455.1 ± 10.45 ${}^{c}$	552.8 ± 10.18 ${}^{a}$	506.0 ± 7.94 ${}^{b}$	3	24.46	< 0.001
Week 3	1027.5 ± 16.39 ${}^{b}$	928.3 ± 16.16 ${}^{d}$	1074.1 ± 15.59 ${}^{a}$	959.0 ± 10.70 ${}^{c}$	3	27.24	< 0.001
Week 4	1617.6 ± 29.4 ${}^{\text{ab}}$	1596.4 ± 29.25 ${}^{\text{bc}}$	1669.8 ± 28.49 ${}^{a}$	1555.2 ± 22.41 ${}^{c}$	3	13.43	< 0.001
Week 5	2196.2 ± 24.66	2160.9 ± 24.66	2214.4 ± 24.66	2155.6 ± 13.18	3	2.19	0.088

${}^{1}$ Data are represented as least square means ± SE of weekly body weight for varying group sizes. ${}^{2}$ ${\text{df}}_{\text{gs}}$ : between group sizes degree of freedom. ${}^{a,b,c,d}$ Group size (GS) means are compared at each age using Fisher's LSD test. Means with different letters in the same row indicate significant differences among GS treatments ( P < 0.05).

The climatic environment data (temperature, humidity, air velocity) of the houses were obtained daily with sensors connected to automation. In the weekly body weight weighing, the birds were caught randomly using catching wire. Two people held this wire at both ends, and after making sure that at least 50 chickens were caught each time, the wire was closed. In this way, 50 chickens (150 chickens in total in each compartment) were weighed randomly from 3 different locations in each pen. The same procedures were followed on the final day of each trial, and individual BWs and body defects were determined. Body defects include footpad dermatitis (FPD), hock burn (HB), and the breast burn-redness (BB) level of 150 chickens (75 male and 75 female) randomly from each group. FPD, HB, and BB levels were determined using a visual scoring system on a 0–3 scale (Welfare Quality, 2009; de Jong et al., 2014; Erensoy et al., 2020b). Litter moisture content was determined after the chickens were slaughtered. Litter samples were collected from three different locations in each group and mixed. Then, 100 g of this mixture was dried at 60 ${}^{\circ }C$ for 48 h and moisture content was measured (Yamak et al., 2016). Data collection and measurement methods for the GS20 000 treatment were performed using the same procedure as the GS6000, GS4000, and GS3000 treatments.

### 2.3 Statistical analysis

All data were analyzed by the generalized linear mixed model (GLMM) procedure of MINITAB (Minitab Inc., UK) software with Gaussian distribution, Version 18.0. Outliers were excluded from the dataset according to the three-sigma rule (De Vries and Reneau, 2010), that is, the data within 3 standard deviations from the mean. The suitability of data for each trait was confirmed using the Shapiro–Wilk test, and they showed a Gaussian distribution ( P > 0.05).

Weekly BW (from 0 to 5 weeks), mortality, and total livability data were subjected to mixed model analysis, using the MIXED procedure by applying the following model:

4 $$Y_{ij}=\mu +\alpha_{i}+\gamma_{j}+\varepsilon_{ij},$$

where $Y_{ij}$ is the observed dependent variable, μ is the overall mean, $\alpha_{i}$ is the fixed effect of group size (3000, 4000, 6000, or 20 000), $\gamma_{j}$ is the block effect (each trial, 1, 2, and 3, is considered as a block), and $\varepsilon_{ij}$ is the residual error. For BW at slaughter, FPD, HB, and BB data, the following model is also applied using MIXED procedure:

5 $$Y_{ijk}=\mu +\alpha_{i}+\beta_{j}+(\alpha \beta {)}_{ij}+\gamma_{k}+\varepsilon_{ijk},$$

where $Y_{ijk}$ is the observed dependent variable, μ is the overall mean, $\alpha_{i}$ is the effect of group size (3000, 4000, 6000, or 20 000), $\beta_{j}$ is the effect of sex, ( $\alpha \beta {)}_{ij}$ is the effect of the interaction between group size treatment and sex, $\gamma_{k}$ is the block effect (each trial, 1, 2, and 3, is considered as a block), and $\varepsilon_{ijk}$ is the residual error. The slaughter age is also added to the model as a covariate to standardize the differences in BWs at different slaughter ages. For body defect traits (FPD, HB, and BB), the GLMM includes the trait BW at slaughter age as a covariable to remove potential biases due to weight-associated effects. The different numbers of pens within the broiler house ( n = 2 in GS6000; n = 3 in GS4000; n = 4 in GS3000) are also used as a random effect for each model.

Average BW, feed intake and FCR values taken from the slaughterhouse were used to calculate the EPEF values for each group size treatment. The ambient temperature, relative humidity, air velocity, and litter moisture content data of each group size houses were subjected to variance analysis. All results were given as least square means with a standard error ( ± SE). Effects were considered to be significant at P < 0.05. Multiple comparisons were performed using Fisher's least significant difference (LSD) test when the significance level was P < 0.05.

## 3 Results

The effects of varying group sizes (GSs) on weekly BW in broilers are shown in Table 3. The chick weights at day-old age were similar among GS treatments and 43.9, 43.6, 43.6, and 43.4 g in the GS3000, GS4000, GS6000, and GS20 000 groups, respectively. GS3000 and GS6000 broilers had higher BW than the GS4000 and GS20 000 chickens at 1 ( P = 0.040) to 2 weeks of age ( P < 0.001). At 3 and 4 weeks of age, GS6000 broilers had the highest BW and GS20 000 broilers had the lowest ( P < 0.001). However, GS treatments did not significantly affect BW of chickens at 5 weeks of age.

**Table 4:** Least square mean and standard error ( ± SE) of body weight ( g ), foot pad dermatitis, hock burn, and breast burn levels in slaughter age broilers reared in varying group sizes ${}^{1}$ .

Group size	Sex	Mean BW at slaughter ${}^{2}$	FPD	HB	BB
GS3000	Female	2360.3 ± 41.28 ${}^{e}$	1.27 ± 0.12	1.18 ± 0.07	1.86 ± 0.05
	Male	2762.5 ± 40.95 ${}^{c}$	1.19 ± 0.12	1.17 ± 0.06	1.83 ± 0.05
GS4000	Female	2417.8 ± 43.92 ${}^{\text{de}}$	1.42 ± 0.12	1.17 ± 0.07	2.17 ± 0.06
	Male	2890.7 ± 44.72 ${}^{b}$	1.58 ± 0.13	1.27 ± 0.08	2.36 ± 0.06
GS6000	Female	2466.9 ± 42.26 ${}^{d}$	1.62 ± 0.12	1.54 ± 0.07	2.38 ± 0.05
	Male	3005.9 ± 43.15 ${}^{a}$	1.59 ± 0.12	1.47 ± 0.07	2.38 ± 0.06
GS20 000	Female	2435.6 ± 41.25 ${}^{d}$	1.34 ± 0.12	1.29 ± 0.07	2.27 ± 0.05
	Male	2887.2 ± 41.56 ${}^{b}$	1.40 ± 0.12	1.38 ± 0.07	2.31 ± 0.05
Effects					
Group size		< 0.001	< 0.001	< 0.001	< 0.001
3000		2561.4 ± 38.58 ${}^{c}$	1.23 ± 0.11 ${}^{c}$	1.17 ± 0.06 ${}^{c}$	1.85 ± 0.04 ${}^{b}$
4000		2654.3 ± 41.98 ${}^{b}$	1.50 ± 0.12 ${}^{\text{ab}}$	1.22 ± 0.07 ${}^{\text{bc}}$	2.26 ± 0.05 ${}^{a}$
6000		2736.4 ± 40.26 ${}^{a}$	1.60 ± 0.11 ${}^{a}$	1.50 ± 0.06 ${}^{a}$	2.38 ± 0.04 ${}^{a}$
20 000		2661.4 ± 39.75 ${}^{b}$	1.37 ± 0.11 ${}^{b}$	1.33 ± 0.06 ${}^{b}$	2.29 ± 0.04 ${}^{a}$
Sex		< 0.001	0.576	0.448	0.248
Female		2420.2 ± 37.03 ${}^{b}$	1.41 ± 0.02	1.29 ± 0.05	2.17 ± 0.03
Male		2886.6 ± 37.13 ${}^{a}$	1.44 ± 0.02	1.32 ± 0.06	2.22 ± 0.03
Interaction		0.007	0.129	0.142	0.123

${}^{1}$ Data are represented as least square means ± SE. F values were 4.01, 14.33, 15.51, and 35.35 for mean BW at slaughter, FPD, HB, and BB, respectively. ${}^{2}$ Slaughter age and mean BW at slaughter age were added to the model as a covariate in order to make an accurate comparison among group size treatments. FPD: foot pad dermatitis; HB: hock burn; BB: breast burn. ${}^{a,b,c,d,e,f}$ Group size (GS) means are compared at each age using Fisher's LSD test. Means with different letters in the same column indicate significant differences among GS ( P < 0.05).

Body weight, footpad dermatitis, hock, and breast burn levels of chickens at slaughter age reared in varying group sizes are given in Table 4. Chickens reared in GS6000 (2736.4 g ) had the highest mean BW at slaughter age ( P < 0.001). GS20 000 and GS4000 chickens showed similar and higher BW than GS3000 group ( P < 0.001). Males were 466.4 g heavier than females ( P < 0.001). Interaction effects on mean BW at slaughter were significant ( P = 0.007), and male broilers in all GS treatments had higher values than females. The male chickens in GS6000 (3005.9 g ) had the highest BW, and GS4000 (2890.7 g ) and GS20 000 (2887.2 g ) males were similar with a higher mean BW at slaughter than in GS3000 (2762.5 g ) males. The mean BWs of females at slaughter were found to be lower than those of males.

As illustrated in Table 4, varying group sizes significantly affected the level of FPD ( P < 0.001, F value = 14.33), HB ( P < 0.001, F value = 15.51), and BB ( P < 0.001, F value = 35.35) in broilers. FPD and HB levels were the highest in GS6000 broilers compared to other group sizes ( P < 0.001). The level of BB was the lowest in GS3000 chickens ( P < 0.001). The levels of FPD, HB, and BB were not affected by sex and interactions (Table 4).

**Figure 1:**
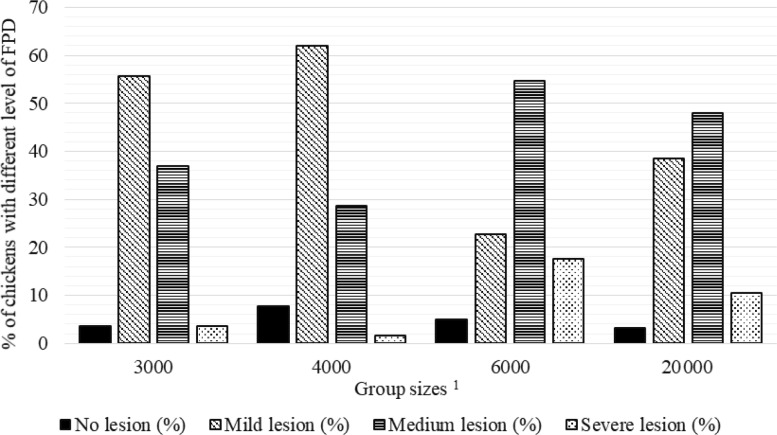
Percentage of chickens with different level of foot pad dermatitis (FPD) in varying group sizes. ${}^{1}$ The 3000, 4000, 6000, and 20 000 numbers represent the number of chickens in the each group size.

**Figure 2:**
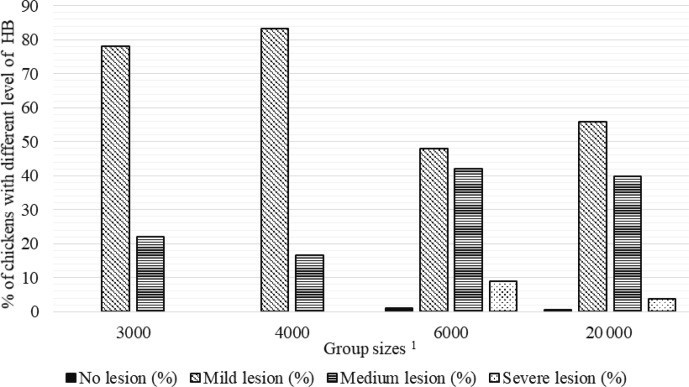
Percentage of chickens with different level of hock burn (HB) in varying group sizes. ${}^{1}$ The 3000, 4000, 6000, and 20 000 numbers represent the number of chickens in the each group size.

**Figure 3:**
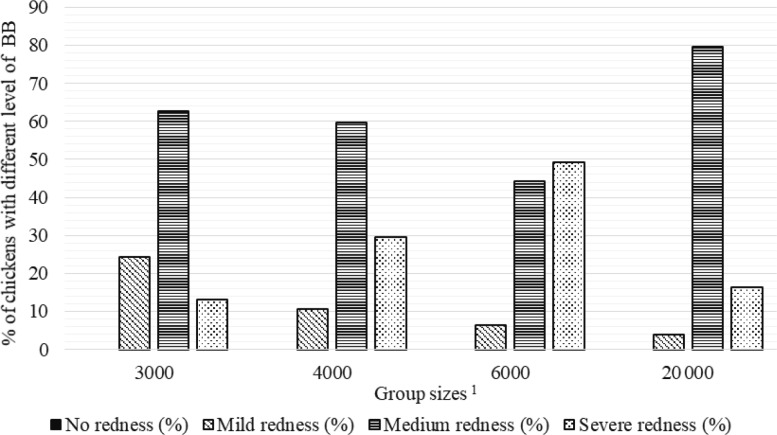
Percentage of chickens with different level of breast burn (BB) in varying group sizes. ${}^{1}$ The 3000, 4000, 6000, and 20 000 numbers represent the number of chickens in the each group size.

**Figure 4:**
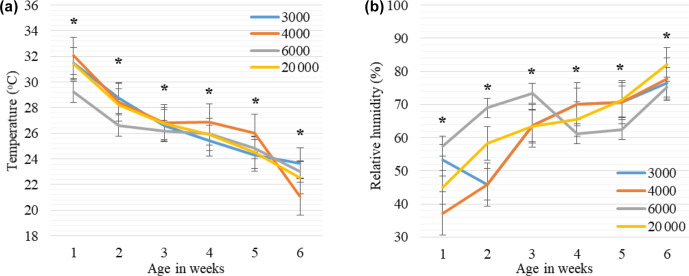
Weekly least square means and standard error ( ± SE) of environmental temperature **(a)** and relative humidity **(b)** changes in houses with varying group sizes. The 3000, 4000, 6000, and 20 000 numbers represent the number of chickens in the each group size. ${}^{*}$ : < 0.005 represents a significance level for differences in ambient temperature ( ${}^{\circ }C$ ) and relative humidity (%) within each week.

Percentages of chickens with different levels of FPD, HB, and BB severity in varying group sizes are shown in Figs. 2–4, respectively. The percentage of broilers with no FPD lesion (7.7 %) and mild level (62.0 %) FPD was the highest in the GS4000, while the percentage of chickens with medium (54.7 %) and severe (17.7 %) FPD lesion was the highest in GS6000 (Fig. 1).

There were no chickens without HB lesions in the GS3000 and GS4000 groups, and 78 % and 83.3 % of the chickens were mild-level HB, respectively. The medium HB was determined in 42 % of GS6000 broilers, and 9 % of them had severe HB at the highest level (Fig. 2).

As seen in Fig. 3, there were no chickens without BB lesions in all groups. The highest percentage of broilers with mild BB (24.3 %) was seen in GS3000, medium BB (79.7 %) in GS20 000, and severe BB (49.3 %) level in the GS6000 group.

**Table 5:** Least square mean and standard error ( ± SE) of ambient temperature, relative humidity, air velocity, and litter moisture in the houses of broilers reared in varying group sizes ${}^{*}$ .

Group size	Ambient	Relative humidity	Air velocity	Litter moisture
	temperature ( ${}^{\circ }$ C)	(%)	( $m\;s^{-1}$ )	(%)
GS3000	26.9 ± 0.22	62.3 ± 1.02	0.20 ± 0.01	34.1 ± 3.19
GS4000	27.0 ± 0.29	60.4 ± 1.44	0.23 ± 0.02	30.5 ± 2.73
GS6000	26.2 ± 0.22	65.7 ± 1.17	0.17 ± 0.02	38.1 ± 2.16
GS20 000	26.7 ± 0.26	63.3 ± 1.30	0.24 ± 0.02	36.4 ± 2.75
P values	0.212	0.055	0.214	0.446

${}^{*}$ Data are represented least square means ± SE of ambient temperature, relative humidity, air velocity, and litter moisture values in varying group sizes.

The mean ambient temperature, relative humidity, air velocity, and litter moisture values in the houses of broilers reared in varying group sizes are provided in Table 5, while weekly trends are shown in Fig. 4. In all groups, the differences between mean temperature, humidity, air velocity, and litter moisture values were insignificant ( P > 0.05). The mean temperatures ranged from 26.2 to 27.0 ${}^{\circ }C$ , relative humidity ranged from 60.4 % to 65.7 %, air velocity ranged from 0.17 to 0.24 $m\;s^{-1}$ , and litter moisture ranged from 30.5 % to 38.1 %.

As seen in Fig. 4a, the weekly mean temperature trends tended to decrease with advancing weeks in each group, but differences within the same week were significant for each week ( P < 0.005). The mean ambient temperatures were lower in the GS6000 house compared to the other groups between 0–3 weeks ( P < 0.005). In the GS4000 house, the mean temperature was higher at 4 and 5 weeks and lower at 6 weeks than the other groups ( P < 0.005). As illustrated in Fig. 4b, the weekly relative humidity trends tended to increase with advancing weeks in the houses of GS4000 and GS20 000 groups. However, the GS3000 and GS6000 houses had fluctuating humidity levels. In the GS6000 house, higher humidity was observed between 0–3 weeks and lower between 4–6 weeks compared to other groups ( P < 0.005).

**Table 6:** Mean slaughter age, body weight, feed intake, feed conversion ratio, and EPEF values at slaughter age in broilers reared in varying group sizes.

Group size	Mean slaughter age ( d )	BW ( g ) ${}^{1}$	FCR ${}^{2}$	Livability (%) ${}^{3}$	EPEF ${}^{4}$
GS3000	39.6	2561.4	1.520	95.2	404.5
GS4000	39.6	2654.3	1.679	90.0	358.8
GS6000	39.6	2736.4	1.531	94.5	425.8
GS20 000	36.6	2661.4	1.772	93.5	354.0

${}^{1}$ Means of BW at slaughter age and livability data are represented as least square means. ${}^{2}$ Feed conversion ratio (FCR) was determined as in Eq. ([Disp-formula Ch1.E1]) and calculated from the observed values. ${}^{3}$ Livability was determined as in Eq. (2). ${}^{4}$ EPEF (European efficiency production factor) was determined as in Eq. (3).

EPEF values obtained from mean slaughter age, BW, FCR, and livability values are provided in Table 6. The GS3000, GS4000, GS6000, and GS20 000 chickens reached BWs of 2561.4, 2654.3, 2736.4, and 2661.4 g at 39.6 d of mean slaughter age (Table 6). The EPEF values were in GS6000 (425.8), GS3000 (404.5), GS4000 (358.8), and GS20 000 (354.0) groups from highest to lowest.

**Figure 5:**
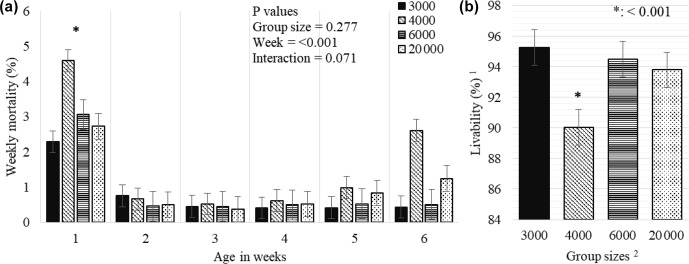
Weekly least square means and standard error ( ± SE) of mortality **(a)** and total livability **(b)** percentages of chickens reared in varying group sizes. ${}^{1}$ Livability was determined as in Eq. (2). ${}^{2}$ The 3000, 4000, 6000, and 20 000 numbers represent the number of chickens in the each group size. ${}^{*}$ : < 0.001 represents a level of significance for weekly total mortality **(a)** among weeks and total livability **(b)** among treatments during 6 weeks of production.

While the GS treatment did not affect the weekly mortality ( P = 0.277), the week effect was significant and a significantly higher mortality was determined in the first week compared to the following weeks ( P < 0.001, Fig. 5a). GS4000 broilers showed lower overall livability (90.0 %) than other groups ( P < 0.001, Fig. 5b).

## 4 Discussion

Commercial fast-growing broilers are generally reared in intensive conditions throughout the world to maximize productive efficiency (Robins and Phillips, 2011). Although this system maximizes production efficiency, it makes sustainability difficult by worsening certain physiological and welfare traits of chickens (Averos and Estevez, 2018) due to stocking densities and group sizes being very high under intensive production. This situation affects the performance, welfare, social behavior, activity, and use of space in broiler chickens (Estevez, 2007; Estevez et al., 2007). The present study focused on comparing the performance, welfare, and some productivity traits of broilers reared in the classic intensive production system (GS20 000) and varying group sizes (GS3000, GS4000, and GS6000).

All GS treatments were started with similar chick weights. At 1 and 2 weeks of age, GS3000 and GS6000 broilers had similar body weights. However, this situation changed at 6 weeks of age and the GS6000 broilers were heavier than others. Our findings were partially consistent with El-Tahawy et al. (2017), who reported that small ( < 10 000 chickens) and medium (11 000 to 30 000 chickens) group sizes showed significantly better slaughter BW than large (31 000 to 50 000 chickens) flocks. Similarly, Ghosh et al. (2012) also determined higher BW at 6 weeks of age in broilers reared in small group than in large ones. Şimşek and Özhan (2015) reported that there was no difference in final BW of broilers reared in different flock sizes of 15 000, 25 000, and 35 000 chickens. In addition, Ali et al. (2012), Rind et al. (2004), and Türkyılmaz (2008) reported that the slaughter BWs of broilers reared in different group sizes were not statistically different. As in our study, the results of relevant studies on the effects of group size on body weights are not always in the same direction and as expected. These results are likely to have potential effects from the different genetics of birds and environmental conditions specific to each study, rather than the direct effect of group size. Nonetheless, it was surprising that broilers reared in a group size of 6000 broilers seem to have higher potential to achieve earlier slaughter BW in our study. Additionally, the BWs at slaughter age for male chickens in all groups were higher than those of females, which implies that males have better growth traits than females due to their biological properties (Schmidt et al., 2009; Erensoy et al., 2020a). While the GS treatment significantly affected the slaughter BW of males, it was generally similar and lower in females than in males due to interaction effects. This suggests that GS affects BW of males rather than females, and GS6000 males had heavier slaughter BW followed by GS4000, GS20 000, and GS3000. Our results showed that rearing male chickens in a group of 6000 chickens resulted in better slaughter weight. While we expected better performance from broilers reared in smaller group sizes (GS3000 and GS4000) in line with Ghosh et al. (2012) and El-Tahawy et al. (2017), the better performance of the GS6000 broilers was surprising and warrants further investigation.

The development of FPD, HB, and BB lesions usually occurs in a similar way. Notably, their level and severity vary depending on the BW, age, and the level of litter moisture (Meluzzi et al., 2008; Kaukonen et al., 2016). Moreover, these ailments represent essential problems in the context of broiler health (Toppel et al., 2019). Poor ventilation management also deteriorates litter quality, resulting in reduced welfare and performance (Kaukonen et al., 2016). Although no differences in litter moisture levels were observed among GS treatments, chickens in GS3000 and GS4000 had significantly lower levels of FPD, HB, and BB than chickens reared in GS6000 and GS20 000 ( P < 0.001). The greater development of body defects in GS6000 broilers might be largely explained by the heavier BW, consistent with Wolanski et al. (2004) and Haslam et al. (2007), because the mean BW at slaughter included in the model for body defects had a significant effect on the level of FPD ( P = 0.012). In our study, HB was less severe than FPD and BB, consistent with van den Oever et al. (2020). The breast area and hocks are only in contact with the litter while sitting; the foot pads are more in contact than the breast and hocks and are also under much pressure due to BW. Although increased lying or resting time in contact with the litter has been reported to increase the incidence of HB (Kjaer et al., 2006; Haslam et al., 2007), no significant direct relationship was found between HB and BW in our study. According to our study results, increased BW pressure contributes to the emergence of FPD. The GS4000, GS6000, and GS20 000 broilers show similar BB, possibly suggesting that the sitting behavior does not change after a certain BW level, in line with van den Oever et al. (2020). However, it is necessary to determine the resting or related behavior measurements for a definite inference.

Rearing chickens in smaller GS (3000 or 4000) decreased the incidence of severe body defects and improved their welfare compared to GS6000 and GS20 000 chickens. In addition to the lower slaughter BW of GS3000 and GS4000 broilers, the possible effect of dividing the house into three or four sections has contributed to improved welfare conditions by preventing unintended bird migration and ensuring a homogeneous chicken distribution (Lacy and Czarick, 1992; Malone, 2004; Czarick and Lacy, 2008). Since the mean values of temperature, humidity, and air velocity were similar among all treatments, this suggests that the environment conditions did not affect the performance, body defects, and productivity of chickens reared in all group sizes.

Regarding first-week mortality, chicks were transferred from a commercial hatchery to our poultry house as soon as they hatched at 0 d . We were unable to control parent stock management, age, vaccination status, or hatching protocols. Torrey et al. (2021) reported that chick deaths within the first 10 d may mostly be related to yolk sac infections or parent stock variables or hatching conditions (Yassin et al., 2009). We speculated that possible aforementioned problems may have affected first-week mortality in our study, regardless of GS effect. While the temperature in our study decreased with age, the humidity increased. These environmental adjustments are required in broiler production farms with advancing age and are also within optimal limits in our study. Therefore, we assumed that environmental conditions had no significant impact on growth characteristics, body defects, or overall livability, as well as EPEF values.

From another perspective, providing uniform ventilation in intensive and large-scale production is not an easy task due to the large house volumes. For this reason, since it may not be possible to provide the same environmental conditions for each chicken in each region of the house, the distribution of chickens in the house is not homogeneous and stocking density may vary from region to region (Lacy and Czarick, 1992; Czarick and Lacy, 2008). Since negative pressure tunnel ventilation was used in the present study, the stocking density of chickens in the GS20 000 house was overcrowded near the fresh air inlets. Dividing the house into several sections (as in our study, four sections in the GS3000, three in the GS4000, and two in the GS6000) to avoid regional overcrowding can prevent possible bird migration (Czarick and Lacy, 2008), but it may result in a lack of activities due to space constraints (Haslam et al., 2007).

European production efficiency factor (EPEF) values, which provide an overall perspective by evaluating the livability, BW, feed efficiency, and mean slaughter age performance values together, are a widely used index that reveals the economic status of production (Huff et al., 2013). Higher EPEF values indicate a better technical performance (Aviagen, 2018). In GS6000 broilers, higher BW and better FCR and livability were effective, especially in achieving the highest EPEF value compared to the other groups. Although the performance was the lowest in GS3000 broilers, better livability and FCR values led to the second highest EPEF values.

In conclusion, rearing broilers in groups is an effective management tool to control untended bird migration in the house and to provide optimal environmental conditions for each bird. Our study showed that rearing in groups of 6000 broilers surprisingly had both better performance and higher overall productivity than other groups; the broilers in this group tended to show more severe body defects. Rearing group size of 3000 broilers promised better welfare status, and overall productivity was secondary. Further studies are needed in which all treatments are tested simultaneously so that possible effects from different growing periods are minimized.

## Supplement

10.5194/aab-65-171-2022-supplement The supplement related to this article is available online at: https://doi.org/10.5194/aab-65-171-2022-supplement.

## Data Availability

All data are available in the Supplement.
