# Supplementary material for: Effects of varying group sizes on performance, body defects, and productivity in broiler chickens
Source: Arch Anim Breed. 2022 May 5;65(2):171–81. doi: 10.5194/aab-65-171-2022 (PMC9097258; doi:10.5194/aab-65-171-2022)
Supplement: The supplement related to this article is available online at: https://doi.org/10.5194/aab-65-171-2022-supplement. [file aab-65-171-supplement.zip › aab-65-171-2022-supplement-title-page.pdf]

Supplement of Arch. Anim. Breed., 65, 171–181, 2022
https://doi.org/10.5194/aab-65-171-2022-supplement
© Author(s) 2022. CC BY 4.0 License.

Open Access
Archives Animal Breeding

Supplement of

Effects of varying group sizes on performance, body defects, and productivity in broiler chickens

Musa Sarıca et al.

Correspondence to: Kadir Erensoy (kadir.erensoy@omu.edu.tr)

- aab-65-171-2022-supplement-title-page.pdf
- Original data
  - 6-weeks BW, FPD, BB.xlsx
  - Altlìk kalitesi.xlsx
  - CA.xlsx
  - EPEF.xlsx
  - Sìcaklìk nem hh.xlsx

The copyright of individual parts of the supplement might differ from the article licence.
